# The contribution of the polio eradication initiative on the operations and outcomes of non-polio public health programs: a survey of programs in the African region

**DOI:** 10.11604/pamj.2018.31.207.17666

**Published:** 2018-11-26

**Authors:** Peter Nsubuga, Ben Masiira, Luka Ibrahim, Nestor Ndakala, Norbert Dongmo

**Affiliations:** 1 Global Public Health Solutions, Atlanta, Georgia, USA

**Keywords:** Polio eradication, public health programs, African region

## Abstract

**Introduction:**

The effect of the Global polio eradication initiative (PEI) on public health programs beyond polio is widely debated. PEI contribution to other health programs has been assessed from the perspective of polio-funded personnel, which may introduce bias as PEI staff are probably more likely to show that they have benefited of other programs. We set out to identify and document how public health programs have benefited from the public health capacity that was provided at the country level as part of the PEI program in a systematic and standardized manner.

**Methods:**

Between July and November 2017, we conducted a mixed-methods cross-sectional study, which combined two methods: a multi-country quantitative survey and a qualitative study. We created a self-administered electronic multi-lingual questionnaire in English, French and Portuguese. The qualitative study, which followed an interim analysis of the quantitative survey, comprised interviews with national and subnational level staff in a few countries.

**Results:**

A total of 127 public health workers from 43 of the 47 countries in the African WHO Region responded online. Most of the respondents 56/127 (42.7%) belonged to the immunization sector and 51/127 (38.9%) belonged to the emergencies and outbreaks sector. Respondents who identified themselves with the immunization (50/64 (78%)) and maternal health program (64/82 (78%)) reported the highest level of greatly benefiting from PEI resources. A total of 78/103 (76%) respondents rated PEI’s contribution data management system to their program very high and high. Of the 127 respondents, the majority 91 (71.6%) reported that the withdrawal of PEI resources would result in a weakening of surveillance for other diseases; 88 (62.9%) reported that there would be inadequate resources to carry out planned activities and 80 (62.9%) reported that there would be poor logistics and transport for implementation of activities. Cameroon, DRC, Nigeria and Uganda participated in the qualitative study. Each country had between 7-8 key informants from the national and sub-national level for a total of 31 key informants. Polio funds and other PEI resources have supported various activities in the ministries of health of the four countries especially IDSR, data management, laboratories and development of the public health workforce. Respondents believed that the infrastructure and processes that PEI has created need to be maintained, along with the workforce and they believed that this was an essential role of their governments with support from the partners.

**Conclusion:**

There is a high awareness of the PEI program in all the countries and at all levels which should be leveraged into improving other child survival activities for example routine immunizations. Future large-scale programs of this nature should be designed to benefit other public health programs beyond the specific program. The public health workforce, surveillance development, data management and laboratory strengthening that have been developed by PEI need to be maintained.

## Introduction

The effect of the Global polio eradication initiative (PEI) on public health programs beyond polio is widely debated [1-4]. However, PEI happens to be one of the only programs that reach vulnerable children in hard to reach areas multiple times a year; indeed many programs have used that reach to tack on several public health interventions [5]. The public health effects of PEI probably differ from the context where there is multiple supplemental immunization activities (SIAs) and national and subnational immunization days (NIDs) to those where polio was interrupted long ago [2, 6]. PEI contribution to other health programs has been assessed from the perspective of polio-funded personnel, which may introduce bias as PEI staff are probably more likely to show that they have benefited other programs. An assessment of what the other programs can define or view as the benefit PEI provided to them is likely to avoid that bias. It is also not clear that other public health programs are aware of what will happen when PEI ends, and there is limited literature that defines this, based on robust scientific methods. Current documentation evaluates how PEI has contributed to programs that are mainly immunization-related - for example in supporting public health surveillance outcomes and supporting routine immunization (RI) and new vaccine introductions [7]. The support and enabling functions (i.e., training, supervision, coordination, communication, other managerial support) of PEI have not been adequately assessed. It is also likely that PEI contributes a lot to operations of WHO Country Offices (WCO) and public health programs in ministries of health (MOH) [8]. An assessment of what benefits programs have received from PEI from the perspective of non-polio public health workers and disease control programs may provide a measure of the gap that will exist when PEI funding ends [9]. It will also help these programs plan for that gap. Additionally, there may be unknown benefits from PEI funds that other programs have obtained. Conversely, PEI may believe that it is benefiting programs that do not perceive this benefit themselves. Based on the above we set out to identify and document how public health programs have benefited from the public health capacity that was provided at the country level as part of the PEI program in a systematic and standardized manner. It is expected that the results of this study will guide the African region on how to utilize the public health capacity from large vertical programs and will also provide lessons to the PEI legacy in the African Region.

## Methods

### Study design

Between July and November 2017, we conducted a mixed-methods cross-sectional study, which combined two methods: a multi-country quantitative survey and a qualitative study. We created a self-administered electronic multi-lingual questionnaire in English, French and Portuguese. The qualitative study, which followed an interim analysis of the quantitative survey, comprised interviews with national and subnational level staff in a few countries. The mixed-methods approach allowed for a triangulated understanding of the how non-polio programs have benefited from the public health capacity that was developed from PEI, from the non-polio programs.

### Quantitative methods

An electronic questionnaire that was optimized for mobile devices and offline use by using SurveyGizmo^TM^, an online survey tool was developed. The questionnaire was available in English, French and Portuguese. A list of survey respondents’ email address, which comprised the following public health professionals was obtained from the World Health Organization’s Regional Office for Africa (WHO-AFRO). The respondents were the following: Routine immunization focal point-WHO (World health organisation) and Expanded Program Immunization (EPI) manager, WHO Country Office Disease Prevention and Control Officer (DPC), Integrated Disease Surveillance and Response (IDSR) focal points-WHO and nationals, Maternal and Child Health focal points-WHO and nationals, WHO-AFRO and WHO-headquarters corporate support for PEI focal points. An email link to the questionnaire in the three languages was sent in July 2017 to the survey respondents. Several reminders were sent to ensure a high response rate. The data were analyzed in MS Excel and Epi Info version 7 (US Centers for Disease Control and Prevention).

### Qualitative methods

In October 2017, four countries (Cameroon, the Democratic Republic of Congo (DRC), Nigeria and Uganda) were assessed focusing on national, provincial or state officials and district or local government officials. Data from WHO Country Office administrators of the four countries were also sought, but not obtained. These four countries provided case studies that allowed the study to obtain granular and explanatory information from the sub-national level.

### Ethical considerations

The study obtained clearance from the WHO-AFRO Ethical Review Committee. Participants’ confidentiality was protected; their contributions were not biased, altered or misrepresented. The identities of participants in the study were protected in the quantitative and qualitative component of the mixed methods study.

## Results

### Description of the respondents in the quantitative study

A total of 127 public health workers from 43 of the 47 countries in the African WHO Region completed all sections of the online questionnaire, although some did not fill out all the variables. The majority 93/123 (76%) were aged 40 to 59 years. Of the respondents, 106/126 (84%) had > 10 years of public health service. The most frequent public health roles of the respondents were program manager 51/127 (40.2%), program advisor 22/127 (28%) and 26/127 (28.3%) were epidemiologists. A total of 66/127 (52%) had a Master of Public Health degree, and 57/127 (44.9%) reported that they had ever been polio-funded staff even if polio did not currently fund them. Most respondents, 113/127 (88.9%) were involved in various polio eradication activities, principally surveillance 91/127 (71.6%), polio supplementary immunization activities (SIA) 87/127 (68.5%) and oral polio vaccination activities 85/127 (66.9%). Most of the respondents 56/127 (42.7%) belonged to the immunization sector and 51/127 (38.9%) belonged to the emergencies and outbreaks sector, and 41/127 (31.3%) belonged to the communicable disease sector (Table 1).

**Table 1:** Public health sector of the respondents PEI Afro Survey 2017

Sector	Number	Percent(n-127)
Immunizations	56	42.7%
Child health	29	22.1%
Maternal health	23	17.6%
Communicable disease	41	31.3%
Emergencies and outbreaks	51	38.9%
Non communicable disease	25	19.1%
Neglected tropical diseases	21	16.0%
Health systems services	21	16.0%
Operations	10	7.6%

Respondents who identified themselves with the immunization (50/64 (78%)) and maternal health program (64/82 (78%)) reported the highest level of greatly benefiting from PEI resources (Table 2). A total of 18/34 (53%) respondents from the child health program and 21/48 (44%) from the communicable disease program reported benefiting greatly from PEI resources. The lowest level of greatly benefiting from PEI resources was from respondents affiliated with the communications program.

**Table 2:** Perception of benefits from polio resources by respondents’ program identification PEI Afro Survey 2017

Program	Count	Percent of the total from the program
Immunizations		
My program has benefited from polio resources greatly	50	78%
My program has benefited from the polio resources somewhat	13	20%
My program has never benefited from polio resources	1	2%
	Total: 64	
Child health		
My program has benefited from polio resources greatly	18	53%
My program has benefited from the polio resources somewhat	13	38%
My program has never benefited from polio resources	3	9%
	Total: 34	
Maternal Health		
My program has benefited from polio resources greatly	64	78%
My program has benefited from the polio resources somewhat	15	18%
My program has never benefited from polio resources	3	4%
	Total: 82	
Communicable Diseases		
My program has benefited from polio resources greatly	21	44%
My program has benefited from the polio resources somewhat	21	44%
My program has never benefited from polio resources	6	13%
	Total: 48	
Noncommunicable Disease		
My program has benefited from polio resources greatly	7	28%
My program has benefited from the polio resources somewhat	11	44%
My program has never benefited from polio resources	7	28%
	Total: 25	
Coordination		
My program has benefited from polio resources greatly	7	28%
My program has benefited from the polio resources somewhat	11	44%
My program has never benefited from polio resources	7	28%
	Total: 25	
Communication		
My program has benefited from polio resources greatly	4	22%
My program has benefited from the polio resources somewhat	12	67%
My program has never benefited from polio resources	2	11%
	Total: 18	
Delivery of intervention commodities		
My program has benefited from polio resources greatly	7	37%
My program has benefited from the polio resources somewhat	8	42%
My program has never benefited from polio resources	4	21%
	Total: 19	

A total of 78/103 (76%) respondents rated PEI’s contribution data management system to their program very high and high (Table 3). This was followed by financial resources rated very high and high by 67/105 (64%) and laboratories rated by 60/97 (62%) of respondents. Only 32/93 (34%) of respondents rated PEI’s contributions to work locations like emergency operations centers as very and high.

**Table 3:** Respondents’ rank of the contributions of the polio resources to their program, PEI Afro Survey 2017

Element	Very highly and Highly	Percent	Not so highly and not at all	Percent	Total
Accountability systems	48	51%	47	49%	95
Governance systems	42	45%	52	55%	94
Data management	78	76%	25	24%	103
Financial resources	67	64%	38	36%	105
ICT equipment	49	51%	47	49%	96
Laboratories	60	62%	37	38%	97
Networks and processes for surveillance	77	75%	26	25%	103
Skilled manpower for public health activities at all levels	77	73%	29	27%	106
Platform for community engagement	57	60%	38	40%	95
Corporate management of the programs and offices	52	51%	49	49%	101
Vehicles and transport for interventions and fieldwork	61	61%	39	39%	100
Vehicles and transport for program management	52	52%	48	48%	100
Work locations like Emergency Operations Centers	32	34%	61	66%	93

Most respondents reported that there would be public health disruptions after the withdrawal of PEI resources (Table 4). A total of 80/118 (68%) respondents agreed that the health system in many African countries would be negatively affected. Additionally, 82/123 (67%) of respondents agreed that data collection and management will be disrupted when PEI resources end. Also, 77/121 (64%) agreed that the Global Vaccination Action Plan (GVAP) targets would not be met after PEI resources end, and 71/121 (59%) respondents believed that there would be a breakdown in disease surveillance performance. However, there was 47/119 (43.2%) level of uncertainty on whether the withdrawal of the PEI resources will affect the achievement of Millennium Development Goals (MDGs) in Africa.

**Table 4:** Opinions about the withdrawal of the human, physical, infrastructure and knowledge resources generated in polio eradication

Element	Agree	Percent	Do not Agree	Percent	Total
Data collection and management will be badly affected	82	67%	41	33%	123
Many public health interventions may not be implemented	61	50%	60	50%	121
Many public health interventions will suffer	75	63%	45	38%	120
The health system in many African countries will be negatively affected	80	68%	38	32%	118
The Millennium Development Goals (MDGs) and Sustainable Development Goals (SDGs) will not be met in the African Region	50	42%	68	58%	118
The public health laboratories will be badly affected	64	54%	55	46%	119
The targets of the Global Vaccine Action Plan (GVAP) may not be met	77	64%	44	36%	121
There will be a breakdown in disease surveillance performance	71	59%	50	41%	121
Supervision and monitoring of other public health programs will be affected	77	63%	45	37%	122
National governments will not be able to sustain the gains made in public health due to their reliance on polio resources	68	56%	54	44%	122
There will be no adverse effect on other public health programs	24	21%	88	79%	112
WHO may not be able to support the countries effectively	60	50%	60	50%	120
It will weaken corporate management of the programs and offices	57	48%	62	52%	119

Out of the 127 respondents, the level of awareness of the programs that had benefited from PEI resources was the highest for data management systems (88, 69.3%), then, networks and processes of surveillance (83, 65.4%), and financial resources (75, 59.1%) (Table 5). The lowest levels of awareness were for work locations like emergency operations centers (23, 18.1%) and accountability and governance systems (39, 30.7%).

**Table 5:** Respondents’ awareness of programs that have benefited from PEI resources, PEI Afro Survey 2017

Programs	Number	Percent(n = 127)
Accountability and governance systems	39	30.7%
Data management systems	88	69.3%
Financial resources	75	59.1%
Information and communications technology equipment	52	40.9%
Laboratories	64	50.4%
Networks and processes for surveillance	83	65.4%
Skilled manpower for public health activities at all levels	71	55.9%
Platforms for community engagement	53	41.7%
Vehicles for interventions	72	56.7%
Vehicles for program management	57	44.9%
Office running costs	48	37.8%
Work locations like emergency operations centers	23	18.1%

Of the 127 respondents, the majority of 91 (71.6%) reported that the withdrawal of PEI resources would result in a weakening of surveillance for other diseases; 88 (62.9%) reported that there would be inadequate resources to carry out planned activities and 80 (62.9%) reported that there would be poor logistics and transport for implementation of activities (Table 6). Only 14 (11%) of the 127 respondents felt that there would be no impact at all from the withdrawal of the PEI resources.

**Table 6:** Respondents’ views on the implication of withdraw of PEI resources: PEI Afro Survey 2017

Opinion	Number	Percent(n = 127)
Inadequate financial resources to carry out planned activities	88	69.3%
Inadequate human resources to carry out planned activities	64	50.4%
Poor logistics and transport for implementation of activities	80	62.9%
Weak program management	46	36.2%
Weak laboratories’ support and performance	59	46.5%
Weak surveillance for other diseases	91	71.6 %
Weak operations	47	37%
Weak corporate management of the programs and offices	42	33.1%
Weak support to countries and other public health programs	58	45.7%
Weak partner coordination and governance	29	22.8%
No implication at all	14	11%

Of the 127 respondents, the majority of 114 (89.1%) reported that country governments at all levels had the responsibility to sustain existing polio funded structures when the PEI funding ended (Table 7). Slightly over half of the respondents (74, 57.8% and 73, 57%) felt that global partners and donors or other public health program should sustain polio funded structures when the PEI funds end.

**Table 7:** Respondents’ opinions on who should sustain the existing polio funded structures, PEI Afro Survey 2017

Opinion on who should sustain polio funded resources	Number	Percent (n= 127)
Government at all levels	114	89.1%
Global partners and donors	73	57%
Public health programs other than polio program	74	57.8%
Non-governmental organizations	39	30.5%
Civil society organizations	47	36.7%
Local communities	60	46.9%
Not sure	3	2.3%

### Results from the qualitative study

Cameroon, DRC, Nigeria and Uganda participated in the qualitative study. Each country had between 7-8 key informants from the national and subnational level for a total of 31 key informants. Polio work was integrated into all levels of the ministries of health in all the four countries. Several of the respondents had participated in PEI activities especially SIAs. Polio being jointly managed with EPI in most cases has supported RI (Routine immunization) and new vaccine introduction. Polio funds and other PEI resources have supported various activities in the ministries of health of the four countries especially IDSR (Integrated Disease Surveillance and Response), data management, laboratories and supported essential ministry of health activities like supervision and outbreak response. Polio funds have supported the training and development of the public health workforce in the four countries. Respondents believed that the infrastructures and processes that PEI has created need to be maintained, along with the workforce and they believed that this was an essential role of their governments with support from the partners. All respondents at all levels were aware that PEI resources were ending soon and they believed that the end of these resources would leave significant gaps in public health activities and this needs adequate planning.

## Discussion

Our study, which is the first ever African continent-wide look at what non-polio programs believe is the benefit from PEI, showed interesting but consistent results between the quantitative survey and the qualitative study. There is a high level of awareness of the PEI program, and most public health workers interviewed had been involved in PEI in one way or another, which shows the considerable effort towards public health capacity development that PEI implemented. Furthermore, IDSR which is the primary strategy that the continent uses for public health surveillance and response, as well as IHR (International health regulation), would not be where it is now without PEI’s investment [10].

The PEI program over the years of its existence has provided the largest resources to any public health intervention on the African continent [11]. Because the enduring success of the gains against polio depends on maintaining a more robust public health system that can conduct surveillance for any new polio cases, immunize children against polio and other childhood illnesses, and respond to polio and other disease outbreaks, it is imperative that the public health system that PEI supported is maintained. In a previous mixed methods study on the impact of PEI on primary health care, there was some evidence of PEI’s support for surveillance and the public health workforce [2]. However, support for surveillance was limited in Nigeria one of the countries that were envolved in this study. In this study, there was a significant evidence that PEI has felt to support other public health programs from the perspective of those programs. Nigeria relied heavily on its PEI program resources when responding to the 2014 Ebola Virus Disease outbreak, and has continued to rely on PEI for aspects of IDSR [12, 13].

One theme from the study is the need to maintain what the public health activities that were implemented by PEI after the PEI resources are exhausted. Most respondents believed that there would be public health disruptions when the funding comes to an end. How this will happen was not clear to the respondents, but they believed that this should be led by the member States, each using the different health partnerships and resources to maintain essential activities that were supported by PEI. One clear adjacency is routine immunization which paradoxically is at low levels in some countries that have done well in PEI-strategies to leverage PEI before it ends to launch more commitment to routine immunization and maternal and child health programs need to be implemented as soon as possible; if this is successful, this will be one legacy for PEI [4].

Our study relied on recalling events that could have occurred in the past especially in countries where polio has now been eliminated; this may have led to a form of recall bias. Also, whereas the quantitative survey had a high response rate, not everybody responded, and it is therefore possible that those who responded have different opinions from those who did not respond. We also sought for but did not obtain data from the WHO Country Offices of the four countries that were included in the qualitative study and therefore there are no results on corporate support of PEI activities or the role of PEI resources in supporting WHO Country Offices operations. However, using mixed methods study allowed for triangulation of the information, particularly in obtaining subnational level public health professionals opinions, enriches the information from the multi-country survey which had national level staff for the most part.

The general objective of the study was to identify and document how public health programs have benefited from the public health capacity that was provided at the country level as part of the PEI program in a systematic and standardized manner. It is expected that the results of this mixed methods study will guide the African region on how to utilize the public health capacity from large vertical programs and will also provide lessons to the PEI legacy in the African Region.

The overall conclusions from the study are the following. Over 150 public health personnel were involved in the mixed methods study, with 42 countries in the quantitative and four in the qualitative study. Almost all the respondents in the mixed methods study were aware of the polio program and the majority were involved in various PEI activities. The commonest PEI activities they were engaged in were SIAs and surveillance. Respondents strongly agreed or agreed that PEI resources were involved in many public health functions. The most frequently reported benefits from PEI by the respondents were networks and processes of surveillance, a skilled workforce, data management and finances. Most respondents reported that the PEI program had supported their work with resources and that PEI was largely integrated with their activities. Respondents indicated that the highest ranking of contributions of PEI was to data management, a skilled workforce, processes for surveillance and the laboratory network. The lowest contribution was attributed to the Emergency Operation Centers (EOCs). Respondents believed that the infrastructure and operations that PEI has created need to be maintained, along with the workforce and they believed that this was an essential role of their governments with support from the partners. All respondents at all levels were aware that PEI resources were ending soon, and they believed that the end of these resources would leave significant gaps in public health activities and this need to be planned for appropriately.

## Conclusion

We conclude and recommend the following: there is a high awareness of the PEI program in all the countries and at all levels which should be leveraged into improving other child survival activities, for example routine immunizations. There have been several benefits from PEI that public health workers are aware of and can identify. Future large-scale programs of this nature should be designed to benefit to other public health programs beyond the specific program. The public health workforce, the surveillance development, the data management and the laboratory strengthening that have been developed by PEI need to be maintained. The onus is on the ministries of health to spearhead this activity. However, specific plans need to be formed soon to avoid anticipated public health gaps when PEI funding ends.

### What is known about this topic

- The polio eradication initiative (PEI) happens to be one of the only programs that reach vulnerable children in hard to reach areas multiple times a year in Africa;
- PEI contribution to other health programs has been assessed from the perspective of polio-funded personnel, which may introduce bias as PEI staff are probably more likely to show that they have benefited other public health programs;
- It is also not clear that other public health programs are aware of what will happen when PEI ends and there is limited literature that defines this, based on robust scientific methods.

### What this study adds

- This study which is the first ever African continent-wide look at what non-polio programs believe is the benefit from PEI showed interesting but consistent results between the quantitative survey and the qualitative study;
- There is a high level of awareness of the PEI program, and most public health workers interviewed had been involved in PEI in one way or another, which shows the considerable effort towards public health capacity development that PEI implemented;
- The Integrated Disease Surveillance and Response strategy which is the primary strategy that the continent uses for public health surveillance and response, as well as the International Health Regulations, would not be where it is now without PEI’s investment.

## Competing interests

The authors declare no competing interests.

## References

[1] Taylor CE, Cutts F, Taylor ME (1997). Ethical dilemmas in current planning for polio eradication. Am J Public Health.

[2] Closser S, Cox K, Parris TM, Landis RM, Justice J, Gopinath R (2014). The impact of polio eradication on routine immunization and primary health care: a mixed-methods study. J Infect Dis.

[3] Levin A, Ram S, Kaddar M (2002). The impact of the global polio eradication initiative on the financing of routine immunization: case studies in Bangladesh, Cote d’Ivoire and Morocco. Bull World Health Organ.

[4] Craig AS, Haydarov R, O’Malley H, Galway M, Dao H, Ngongo N (2017). The public health legacy of polio eradication in Africa. J Infect Dis.

[5] Cochi SL, Freeman A, Guirguis S, Jafari H, Aylward B (2014). Global polio eradication initiative: lessons learned and legacy. J Infect Dis.

[6] Okeibunor J, Nshimirimana D, Nsubuga P, Mutabaruka E, Tapsoba L, Ghali E, Kabir SH, Gassasira A, Mihigo R, Mkanda P (2016). Documentation of polio eradication initiative best practices: experience from WHO African Region. Vaccine.

[7] Anya BM, Moturi E, Aschalew T, Carole Tevi-Benissan M, Akanmori BD, Poy AN, Mbulu KL, Okeibunor J, Mihigo R, Zawaira F (2016). Contribution of polio eradication initiative to strengthening routine immunization: lessons learnt in the WHO African region. Vaccine.

[8] Loevinsohn B, Aylward B, Steinglass R, Ogden E, Goodman T, Melgaard B (2002). Impact of targeted programs on health systems: a case study of the polio eradication initiative. Am J Public Health.

[9] Patel M, Cochi S (2017). Addressing the challenges and opportunities of the polio endgame: lessons for the Future. J Infect Dis.

[10] Kasolo F, Yoti Z, Bakyaita N, Gaturuku P, Katz R, Fischer JE (2013). IDSR as a platform for implementing IHR in African countries. Biosecurity Bioterrorism Biodefense Strategy Pract Sci.

[11] Rutter PD, Hinman AR, Hegg L, King D, Sosler S, Swezy V (2017). Transition Planning For After Polio Eradication. J Infect Dis.

[12] WHO. Regional Office for Africa Polio personnel support Lassa fever response in Nigeria for rapid containment of an unprecedented outbreak.

[13] Vaz RG, Mkanda P, Banda R, Komkech W, Ekundare-Famiyesin OO, Onyibe R (2016). The role of the polio program infrastructure in response to Ebola virus disease outbreak in Nigeria 2014. J Infect Dis.

